# The transdermal cream of Formestane anti-breast cancer by controlling PI3K-Akt pathway and the tumor immune microenvironment

**DOI:** 10.3389/fimmu.2023.1041525

**Published:** 2023-03-28

**Authors:** Lanyang Gao, Lei Zhu, Chen Shen, Xiaoming Hou, Youyou Chen, Linglin Zou, Huiyan Qiang, Alexander T. Teichmann, Wenguang Fu, Yao Luo

**Affiliations:** ^1^ Academician (Expert) Workstation of Sichuan Province, The Affiliated Hospital of Southwest Medical University, Southwest Medical University, Luzhou, China; ^2^ West China Hospital, Sichuan University, Chengdu, China; ^3^ Sichuan Provincial Center for Gynaecology and Breast Disease, The Affiliated Hospital of Southwest Medical University, Southwest Medical University, Luzhou, China

**Keywords:** Formestane cream, DMBA-induced mammary cancer, PI3K-Akt signaling pathway, tumor immune microenvironment, RNA transcriptome sequencing

## Abstract

**Background:**

Treatment of ER^+^ breast cancer with intramuscular formulation of Formestane (4-OHA) shrinks the tumor within weeks. Since the tedious way of intramuscular administration and side effects are not suited for adjuvant treatment, Formestane was withdrawn from the market. A new transdermal formulation of 4-OHA cream may overcome the defects and retain the effect of shrinking the breast cancer tumor. However, the effects of 4-OHA cream on breast cancer need further confirmatory studies.

**Methods:**

In this work, *in vivo*, the influence of 4-OHA cream on breast cancer was evaluated using the mode of 7,12-dimethylbenz(a)anthracene (DMBA) induced rat mammary cancer. We explored the common molecule mechanisms of action of 4-OHA cream and its injection formulation on breast cancer through RNA- sequencing-based transcriptome analysis and several biochemical experiments.

**Results:**

The results showed that the cream substantially reduced the entire quantity, size, and volum of tumors in DMBA-treated rats consistent with 4-OHA injection, and indicated that there were comprehensive signals involved in 4-OHA antitumor activity, such as ECM-receptor interaction, focal adhesion, PI3K-Akt signaling pathway, and proteoglycans in cancer. In addition, we observed that both 4-OHA formulations could enhance immune infiltration, especially CD8^+^ T cells, B cells, natural killer cells, and macrophages infiltration, in the DMBA-induced mammary tumor tissues. The antitumor effects of 4-OHA partly depended on these immune cells.

**Conclusion:**

4-OHA cream could inhibit breast cancer growth as its injection formulation and may provide a new way for neoadjuvant treatment of ER^+^ breast cancer.

## 1 Introduction

More than 50% of breast cancers are estrogen receptor alpha (ERα)-positive and their development and growth depend on estrogens (1, 2). Consequently, the major treatment strategy is deprivation of estradiol (E2). In postmenopausal women, estrogen deprivation is achieved by inhibiting aromatase, which is a key enzyme of estrogen synthesis from adrenal precursor molecules (3). 4-hydroxyandrostenedione (4-OHA), named Formestane, is the first specific steroidal aromatase inhibitor (AI) in clinical use (4). Its intramuscular depot preparation (Lentaron^®^Depot) is administered at a single dose of 250 mg/patient/application once every 2 weeks. High serum concentrations of the aromatase inhibitor (AI) are aimed at taking the desired action in the tumor, and exploring whether it would lead to systemic side effects by both reducing the systemic estrogen levels and inhibiting the autocrine production of estrogens. Unfortunately, because the tedious way of administration and side effects are not suited for adjuvant treatment, Lentaron was withdrawn from the market. Nevertheless, clinical trials have found that 4-OHA could be still effective when the tumor showed a relapse in spite of thoroughly removing the E2 by nonsteroidal AIs (5–7). This clinical effect must be due to a mechanism independent from the deprivation of E2. To reuse the clinical benefits of 4-OHA on mammary carcinoma and overcome its disadvantages, Heinrich Wieland and his colleagues developed a new formulation of 4-OHA cream which could be topically applied to the mammary gland and then penetrate through the skin and concentrate in the fatty tissue (8). The cream was clinically evaluated by Savetherapeutics^®^, a Germany-based biotech company. Four studies were conducted, in which resorption, tolerability, and efficacy of the topically applied formestane were shown. However, these promising results need further confirmatory studies to finally achieve market authorization for the transdermal formestane cream.

**Figure 1:**
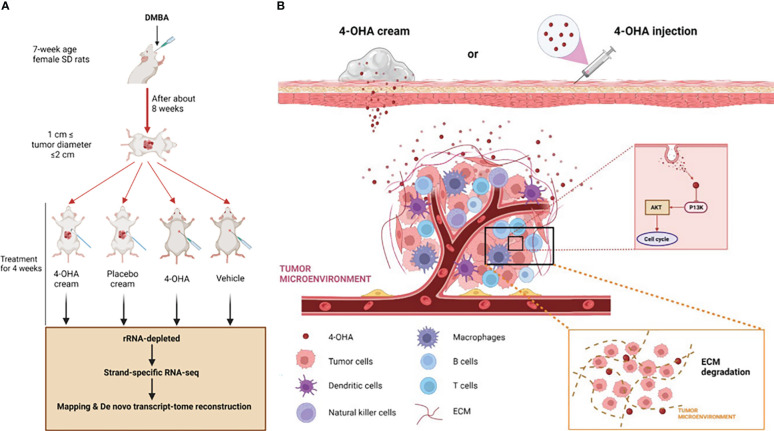
Study design and mechanism of 4-OHA cream anti-breast cancer. Study design. Mammary tumors were initiated with DMBA at PND 49, and when the first tumor per rat reached a size of 1.5 cm in diameter but less than 2 cm, 4-OHA was given to the rats as indicated in the Materials and Methods. **(A)** Tumors responding to 4-OHA were harvested for RNA-seq. **(B)** The mechanism of action of 4-OHA cream.

Brodie et al. had previously shown that 4-OHA markedly shrank dimethylbenz(a)anthracene (DMBA)-induced mammary carcinoma in rats (9). DMBA-induced tumors of rats were the most widely used *in vivo* model of breast cancer (3, 10–12). Therefore, in the study reported here, we also used this model to evaluate the effects of 4-OHA cream on breast cancer (**Figure 1A**). In addition, the effects of 4-OHA on the immune microenvironment in rats’ breast cancer model were assessed as well. Moreover, we clarified the underlying mechanisms of 4-OHA action on breast cancer (**Figure 1B**). This study is the first report to identify the global gene expression profile and the intra-tumor immune landscape of DMBA-induced mammary tumor after treatment with 4-OHA.

## 2 Materials and methods

### 2.1 Animal

Female Sprague-Dawley (SD) rats were supplied by Chengdu dashuo experimental animal Co. Ltd. Rats are housed at five per cage under a regimen of 14 h of light and 10 h of darkness (lights on at 05:00 a.m.). Animals received rat chow and water *ad libitum*. The animal experiments were performed under the ‘Guideline for Care and Use of Experimental Animals’ and were approved by the Institutional Review Board (or Ethics Committee) of The Affiliated Hospital of Southwest Medical University, Southwest Medical University (protocol code 201903-37 and date of approval 2019-03-05).

### 2.2 Mammary tumor model

Six-week-old female SD rats, weighing approximately 150 g, were used in this study. Mammary carcinomas were induced by two oral administrations of DMBA (Sigma Chemical Co.) at a total dose of 20 mg/body at 6 and 7 weeks of age. Starting 40 days after DMBA treatment, animals were examined weekly by palpation; the rats with at least one tumor reaching 1.5 cm in diameter were placed sequentially into experimental groups (10 rats/test group). The body weights of the rats were also measured weekly. Animals with no tumors were discarded 150 days after DMBA treatment. The two perpendicular tumor axes were measured with calipers twice a week. Tumor volume was calculated by the formula *d* ^2^ × *D*/2, where d is the minimal and *D* is the maximal diameter.

### 2.3 Drug and treatment schedule

4-OHA (Sigma Chemical Co.) was dissolved in benzyl alcohol and diluted in peanut oil when given subcutaneously (s.c.) (defined as 4-OHA injection) or made into cream as the formulation of the patent (US 20030092693A1) (designate as 4-OHA cream). Rats were then divided into four groups of 10 animals each. Rats in group A received 1 ml/kg of peanut oil as subcutaneous vehicle daily; rats of group B were applied transdermally with 1 ml of placebo cream twice daily; rats in group C were treated subcutaneously with 1 ml/kg of oil suspension of 4-OHA (50 mg/kg/day) twice daily; group D was administered transdermally with 1 ml of 2.5% 4-OHA cream twice daily. All treatments were maintained for 4 weeks. The doses chosen for testing the antitumor effect of 4-OHA in this study were based on Brodie’s previous studies (13) and the patent of 4-OHA ointment. Tumor growth was expressed as a percent value to the initial tumor volume, measured on the first day of treatment and taken as 100%. At the end of the treatment period, the rats were euthanized with cervical dislocation; tumors were removed and measured. The tissues were removed from fat and necrotic areas, weighed, and stored at -80° or placed in 10% buffered formalin solution until assayed.

### 2.4 UPLC analysis of 4-OHA in cream chemical components

4-OHA cream 10 mg was ultrasonically extracted with 10 ml acetonitrile for 20 min following matrix impurity removal with saturated sodium chloride. The extract was evenly distributed to a 10 ml volumetric flask and the volume was adjusted to 10 ml with methanol to obtain a 1 mg/ml sample solution. After filtration with a 0.22 μm filter membrane, sample injection was determined. UPLC was then used to analyze the sample solution to quantify the content of 4-OHA. The samples were subjected to UPLC analysis on a C18 column (2.1 mm i.d × 100 mm length, 1.6 μm) by a single injection of 5 μl detected at 278 nm; isometric mobile phase: Water (A); Acetonitrile (B) = 50:50; column temperature: 30°C; flow rate: 0.3 ml/min. 4-OHA was identified by their characteristic absorption spectra and their typical retention time corresponding to its standards. The concentration of 4-OHA in cream was determined using a four-level calibration curve based on a concentration series at 10, 20, 30, and 40 μg/ml. The calibration curve showed good linearity (correlation coefficients >0.999).

### 2.5 Cell viability

Cell viability was evaluated by MTT assay. Briefly, MCF-7 and ZR-75-1 cells were seeded in a 96-well culture plate (1000 cells/well). After 24 h, cells were treated by 4-OHA at a dose of 1 μM or left untreated in a 5% CO_2_ incubator for the indicated time. The incubation time and the concentrations of compounds were used based on previous studies (14). At the end of incubation, 10 µl of 5 mg/ml MTT solution was added to each well. After a 3-h incubation, 100 μl DMSO was added to dissolve the resultant purple formazan crystals. The absorbance was measured at 570 nm on a Thermo Scientific Varioskan Flash Multimode Reader.

### 2.6 Colony formation assay

About 2 × 10^3^ breast cancer cells were seeded in six-well plates and exposed to 1 μM 4-OHA in a 5% CO_2_ incubator. After 7 days, cells were fixed with 4% polyoxymethylene and stained with crystal violet. The images for colony formation were recorded by a high-resolution scanner.

### 2.7 Cell cycle

The cell cycle was measured by flow cytometry assays. Breast cancer cells (2 × 10^5^ cells/well) were plated in a 6-well plate and treated with DMSO, or 4-OHA (1 μM) for 72 h. For cell cycle analysis, cells were harvested with trypsin without EDTA to obtain single-cell suspensions and then washed once with ice-cold 1× PBS. After that, cells were fixed overnight in 70% ice-cold ethanol. The fixed cells were washed once with ice-cold 1× PBS, stained with PI solution (50 mg/ml PI, 0.1% NP-40, 0.1% sodium citrate, 0.1% Triton X-100), and then analyzed on a FACS Calibur (BD Biosciences).

### 2.8 RT-PCR and qRT-PCR

Total RNAs were isolated from DMBA-induced tumor tissues of rats or cells using TRIzol reagent (Life Technologies). cDNAs were synthesized by M-MLV reverse transcriptase (Life Technologies) as the manufacturer’s instructions, followed by real-time PCR using SYBR Green mix. Oligonucleotide sequences are listed in **Supplementary Table 1**, **2**.

### 2.9 Western Blot Analysis

Total proteins were extracted in RIPA buffer (25 mM Tris·HCl, pH 7.6, 150 mM NaCl, 1% Nonidet P-40, 1% sodium deoxycholate, 0.1% SDS, 1× phosphotase inhibitor and protease inhibitor cocktail. The resultant proteins in the supernatants were stored at -80°C or directly subjected to SDS-PAGE. Proteins were resolved on SDS-PAGE gels and transferred onto PVDF membranes. The membrane was blocked for 1 h at room temperature in 5% non-fat dry milk or bovine serum albumin (BSA) in TBST buffer (20 mM Tris-HCl, pH 7.5,150 mM NaCl, and 0.1% NP-40), then incubated with the primary antibody at 4°C overnight, washed, and incubated with horseradish peroxidase-conjugated secondary antibody for 1 h. The signals were detected using ECL reagents (Life Technologies). The antibodies used are shown in **Supplementary Table 3**.

### 2.10 Immunohistochemistry and immunofluorescence

Tumor tissues obtained from the rat bearing DMBA-induced mammary carcinoma were subjected to immunohistological analysis. Immunohistochemistry was performed as previously described (15). The tissue sections were blocked by goat serum and incubated with primary antibody (1:200) and then incubated with biotinylated secondary antibody (1:200). Finally, the ImageJ software was used to analyze the data. For IF, sections were incubated with rat anti-mouse CD8, CD19, and CD56/CD16 antibodies, followed by staining with goat anti-rat (Abcam, ab150088) antibodies. DAPI (Invitrogen) was added to counterstain the nuclei. Finally, images were acquired by a slice section scanner (Pannoramic DESK) scanning microscope system and analyzed using the CaseViewer software. The antibodies used are shown in **Supplementary Table 3**.

### 2.11 Flow cytometry analysis

Single-cell suspensions were prepared from tumor tissue homogenates. Contaminated red blood cells were hemolyzed using ammonium chloride solution (IMGENEX). The resulting single-cell suspensions were incubated with the Abs for 30 minutes on ice. Isotype-matched control Igs were used to detect the nonspecific binding of Ig in the samples. The stained cells were analyzed on a CytoFLEX S system (Beckman Coulter, Brea, CA), and the obtained data were analyzed using the FlowJo_v10 software.

### 2.12 Subacute toxicity assessment

The safety of 4-OHA cream *in vivo* was evaluated by serum biochemical analysis, H&E staining. Briefly, following topical administration of 4-OHA cream or placebo cream in SD rats, the peripheral blood samples were collected using heparin and centrifuged for serum biochemical analysis. The tissue samples were simultaneously excised and fixed in 10% buffered formalin. The ALT, AST, TBIL, ALB, BUN, and total protein analyses in serum were performed using blood chemistry assay kits (BioAssay Systems). The tissue samples were embedded in paraffin, then cut into 5-μm-thick paraffin sections and subsequently placed on a glass slide. The slides were stained with H&E. Finally, images were acquired by a slice section scanner (Pannoramic DESK).

### 2.13 RNA Transcriptome sequencing

RNAs 3 μg were used to generate a sequencing library using NEBNext Ultra™ RNA Library Prep Kit for Illumina (New England BioLabs) as the manufacturer’s instructions. Then PCR was performed with Phusion High-Fidelity DNA polymerase, Universal PCR primers, and Index (X) Primer, and the resultant PCR products were purified (AMPure XP system) and library quality was assessed on the Agilent Bioanalyzer 2100 system. The clustering of the index-coded samples was performed on a cBot Cluster Generation System using TruSeq PE Cluster Kit v3-cBot-HS (Illumia) according to the manufacturer’s recommendations. After cluster generation, the library preparations were sequenced on an Illumina Hiseq 2500 platform.

### 2.14 Statistical analysis

The data are presented as the mean ± SD of at least three independent experiments. Statistical analysis among groups was conducted using the two-tailed Student’s *t*-test and one-way analysis of variance on GraphPad Prism (GraphPad Software). The *p*-values <0.05 were considered statistically significant and marked with an asterisk (*, *p* < 0.05; **, *p* < 0.01; ***, *p* < 0.001).

## 3 Results

### 3.1 Effects of 4-OHA cream on breast cancer

**Figure 2:**
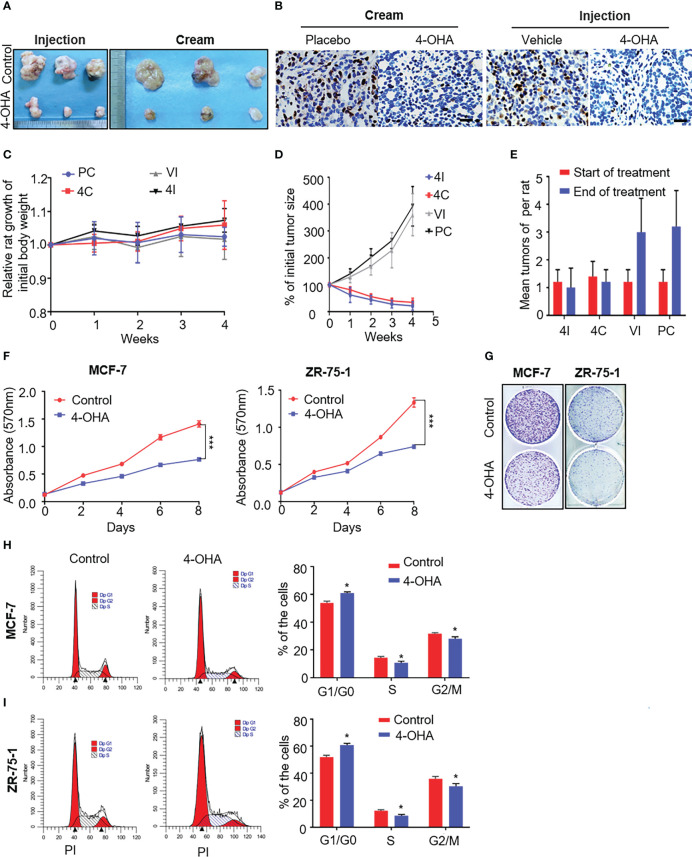
The effects of 4-OHA cream on breast cancer growth. At an age of 50–54 days, female Sprague-Dawley rats were dosed intragastrically with 20 mg DMBA. Animals were selected for experiments when at least one tumor per rat had reached a diameter of 1.5 cm but less than 2 cm. The skin covering the tumor was shaved and the animals were divided into four groups. In 4-OHA and placebo cream groups, tumors were treated by direct application of the cream. **(A–E)** Tumors in the 4-OHA injection group were treated with twice-daily s.c. injections of 1 ml/kg of 4-OHA (50 mg/kg/day), and control animals received injections of the 4-OHA-suspending vehicle (peanut oil) at the same time. **(A)** Representative images of the tumors. **(B)** Expression of the proliferation marker Ki67 in tumor tissues was measured by immunohistochemistry. Scale bar, 20 μm. **(C)** Rat weight evolution during the experiment. The values are expressed as the mean. **(D)** Tumor growth curve. Average number of tumor nodules before and after treatment. **(E)** The data represent six sets of independent experiments and are shown as the means ± SD. *p < 0.05 vs. control group. The effects of 4-OHA on the growth of the cells. **(F–I)** Cells were cultured in 10% PRF-CT with E2 1 nM for 3 days before the experiment. **(F, G)** Cells were seeded in 96-well or six-well plates and 24 h later they were exposed to 1 μM of 4-OHA for 8 days. MTT **(F)** and Colony formation **(G)** assay were performed. Analysis of the cell cycle of MCF-7 **(H)** and ZR-75-1 **(I)** by flow cytometry as described in the Materials and Methods. Data represent a mean ± SEM of three independent experiments, each in triplicate; bars, SEM. *p ≤ 0.05 vs. control. control. PC, Placebo cream; 4C, 4-OHA cream; VI, vehicle; 4I, 4-OHA injection.

Previous studies had shown that 4-OHA given s.c. to rats markedly shrank DMBA-induced mammary carcinoma (9, 16). To investigate whether 4-OHA cream had a similar effect to 4-OHA injection on breast cancer, we established a model of DMBA-induced mammary carcinoma in SD rats, developed the 4-OHA cream based on the formulation of patent, and assessed the antitumor efficacy of 4-OHA cream (**Figure 2** and **Supplementary Figure 1**).

Starting 60 days after DMBA treatment, mammary tumors were successfully induced as shown in **Supplementary Figures 1A–C**. Neoplasias occur with a frequency of >90% (data not shown). DMBA-induced mammary cancer had been proved to be estrogen and progesterone receptor positive (9). Similarly, our immunohistochemical results showed to be ER- and PR-positive in DMBA-induced tumors (**Supplementary** **Figure 1C**). Then, we used this model to repeat Brodie’s results that 4-OHA at a dose of 50 mg/kg per day could diminish tumors by more than half. Likewise, the 4-OHA cream caused a significant regression of tumors (**Figures 2A, D**, and **Supplementary** **Figure 1D**). Immunohistochemical results showed that the proliferation marker Ki67 was reduced by 4-OHA cream or injection as compared with control (**Figure 2B**). The average number of tumor was 1.25 ± 0.15 tumors per rat at the beginning of the treatment for all groups. Values of 3.0 ± 1.2, 3.2 ± 1.3, 1.0 ± 0.7, and 1.2 ± 0.4 were found after 28 days of treatment with vehicle, placebo cream, 4-OHA injection, and 4-OHA cream, respectively. Both 4-OHA dosage forms significantly decreased the number of new tumors at the end of the experiment compared to their respective control (**Figure 2E**). The weight of rats in drug groups increased slightly after 4 weeks of treatment (**Figure 2C**). Subsequently, to confirm that the antitumor effects of the cream derived from the compound of 4-OHA, we performed UPLC to detect the concentration of active ingredients in the cream. The main peak of the cream was identical to the peak of a single compound of 4-OHA (**Supplementary Figure 2A**). Then, according to the calibration curve, the 4-OHA content of the cream was determined to be 2.5%–2.7% (**Supplementary** **Figure 2B**). In consequence, these findings indicated that 4-OHA cream worked in tumors as 4-OHA injection.

Additionally, as shown in **Figures 2F, G**, 4-OHA inhibited the proliferation of MCF-7 and ZR-75-1 cells at a dose of 1 μM. This antiproliferative action of 4-OHA correlated well with the results of the cell-cycle analysis in breast cancer cells. 4-OHA significantly arrested the cell cycle, increasing the portion of MCF-7 and ZR-75-1 cells in the G1 phase and decreasing the portion in the S phase (**Figures 2H, I**), which further demonstrated that the compound of 4-OHA we used worked. In summary, the above results supported the notion that 4-OHA could work in breast cancer through transdermal absorption.

### 3.2 Safety evaluation of 4-OHA cream

**Figure 3:**
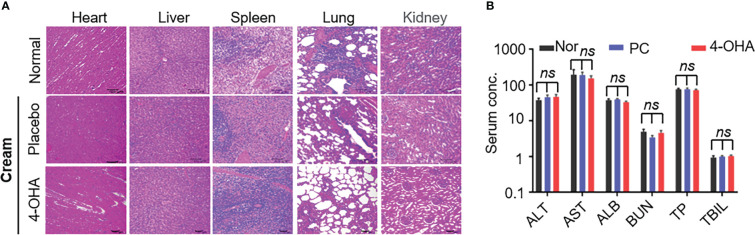
Safety evaluation of 4-OHA. Seven-week-old female SD rats were topically applied with cream at a dose of 10 g/kg. **(A)** H&E staining of main organs in SD rats after treatment with placebo or 4-OHA cream, or without treatment. **(B)** Serum biochemical parameters, including ALT, AST, TBIL, ALB, BUN, and TP, in rats treated with or without cream. Nor, normal; PC, placebo cream; 4-OHA, 4-OHA cream. There is no significance between the group of placebo cream or 4-OHA cream and normal group. *p* > 0.05, *ns*. ns means no significant difference.

*In vivo*, pathological studies in SD rats were performed to evaluate 4-OHA cream safety. The female SD rats were topically applied with placebo cream, 4-OHA cream, or without treatment. Because 1 g/kg was the efficacious dose of 4-OHA cream to suppress DMBA-induced tumor growth in rats (14), the high dose of 10 g/kg was used to evaluate 4-OHA cream toxicity. The serum biochemistry data showed that 4-OHA cream had no hematological or liver toxicity (**Figure 3B**). The H&E staining indicated that there were no obvious necrotic cells or tissues in the major organs, including heart, liver, spleen, lung, and kidney (**Figure 3A**). Moreover, the side effects (vomiting, diarrhea, and significant decrease in rat body weight) were not observed in rats (data not shown). Thus, 4-OHA cream at a 10 g/kg dose was safe.

### 3.3 RNA-Seq gene expression profiling in DMBA-induced tumor treated with 4-OHA cream

**Figure 4:**
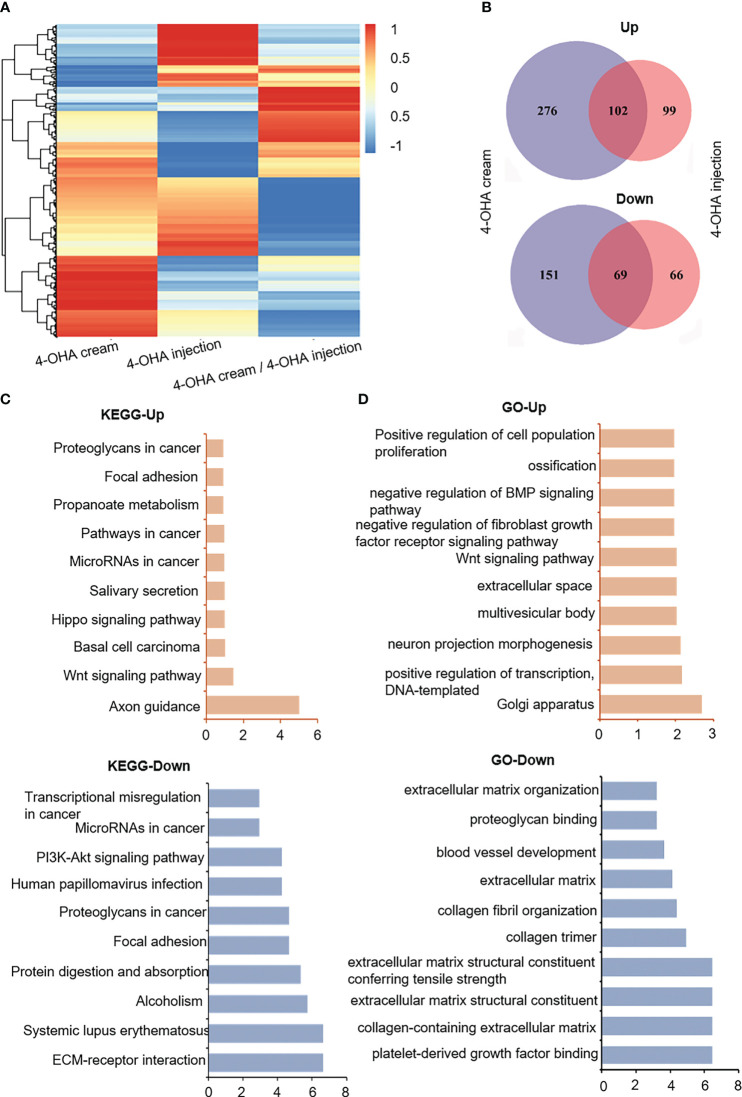
RNA-seq gene expression analysis of DMBA-induced mammary tumors treated with or without 4-OHA. **(A)** Heatmap of detected genes differentially expressed in the control and experimental group. **(B)** Venn diagram of 4-OHA-induced up-regulated or down-regulated DEGs in DMBA-induced tumors treated by 4-OHA cream or 4-OHA injection formulation. Kyoto Encyclopedia of Genes and Genomes (KEGG) **(C)** and Gene Ontology (GO) term **(D)** analysis of common differentially expressed genes between 4-OHA cream and 4-OHA injection group.

To understand the molecular mechanism of action of 4-OHA cream anti-breast cancer, we used high-throughput sequencing technology to determine the differentially expressed genes (DEGs) in the experimental vs. control group. As shown in **Figures 4A, B**, 4-OHA cream caused 378 up-regulated genes (normalized FPKM of experimental group ≥40) and 220 down-regulated genes (normalized FPKM of control ≥40) compared to its control by ≥2-fold (i.e. average |log2 (fold-change)| ≥2). 4-OHA injection vs. vehicle had 201 up-regulated genes and 135 down-regulated genes. Hierarchical clustering based on differentially expressed RNA transcripts (**Figure 4A**) revealed clear clustering of 4-OHA-treated DMBA-induced tumors from the control. Venn diagram showed that 102 up-regulated genes and 69 down-regulated genes were shared by 4-OHA cream and injection (**Figure 4B**), and the remaining DEGs were unique to each individual.

To further explore the biological implications of the common DEGs, the Kyoto Encyclopedia of Genes and Genomes (KEGG) pathway enrichment analysis and Gene Ontology (GO) term analysis were performed with these common DEGs, and the results were shown in **Figures 4C, D**. For KEGG term analysis, it demonstrated that 4-OHA cream as its injection had significant effects on ECM-receptor interaction, focal adhesion, PI3K-Akt signaling pathway, and axon guidance (**Figure 4C**). In the GO term analysis, as expected, DEGs were mainly related to extracellular matrix (ECM) structural components (**Figure 4D**), which have been reported to be associated with PI3K-Akt signaling pathway and tumor proliferation (17).

### 3.4 The effects of 4-OHA on extracellular matrix and PI3K-Akt signaling pathway

**Figure 5:**
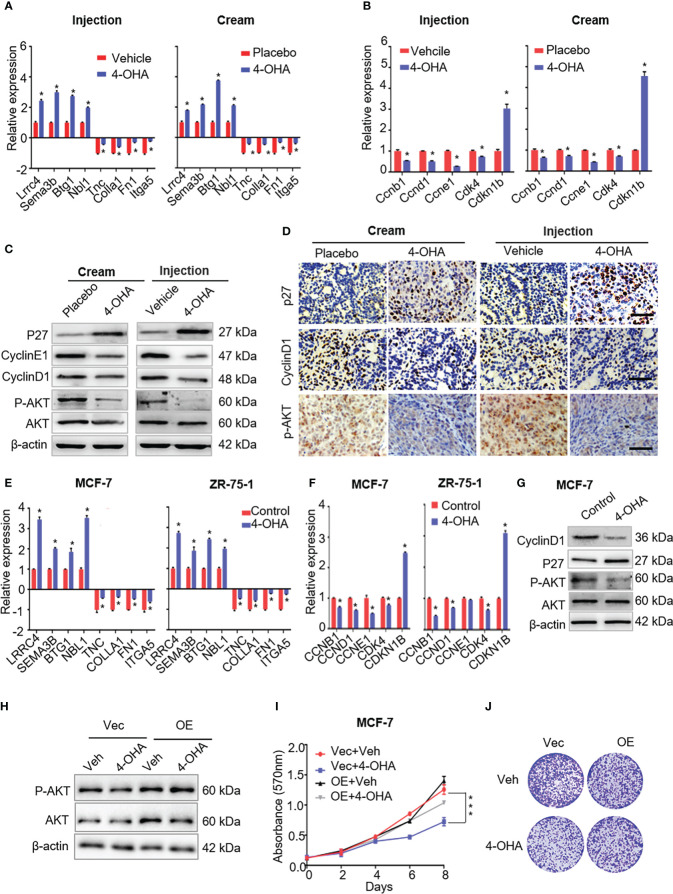
The effects of 4-OHA on extracellular matrix, cell cycle, and PI3K-Akt signaling pathways related genes or proteins. **(A)** The qRT-PCR was carried out to validate indicated DEGs in both 4-OHA cream and 4-OHA injection groups. **(B)** Representative levels of cell cycle-related genes by qRT-PCR. The key protein levels of the cell cycle and the PI3K-Akt signaling pathways were analyzed by western blotting **(C)** and immunohistochemistry **(D)** after 4-OHA treatment in DMBA-induced breast cancer tumors. Scale bar, 50 μm. **(E–G)** The expression levels of cell cycle, PI3K-Akt signaling pathways related genes, and tumor suppressors by qRT-PCR and western blotting in MCF-7 and ZR-75-A cells exposed to 1 μM of 4-OHA for 72 h. **(H–J)**Following Akt overexpression in MCF-7 cells, MTT **(I)** and Colony formation **(J)** assay were performed. The protein expression of Akt and P-AkT were analyzed by western blotting **(H)**. Vec, Vector; OE, overexpression; Veh, Vehicle. Data are represented as the mean ± SEM of three independent experiments, each in triplicate; bars, SEM. ***p ≤ 0.001, *p ≤ 0.05 vs. control.

It is well known that 4-OHA is an aromatase inhibitor that can block estrogen biosynthesis (18). Estrogen activity can modulate components of the ECM in the tumor microenvironment, upregulating transcripts of *COL1A1*, and several matricellular proteins such as *TNC*, *FN1*, and *POSTN* (19). As shown in **Figure 5A**, both 4-OHA treatment methods downregulated transcripts of ECM-related genes in DMBA-induce tumors, such as *Col1a1*, *Tnc*, *Fn1*, and *Itga5*. Meanwhile, the expression levels of these genes in MCF-7 and ZR-75-1 cells were downregulated by 4-OHA at a dose of 1 μM, which suggested that 4-OHA could cause an extracellular matrix remodeling of ER+ breast cancer cells.

Multiple studies have demonstrated remodeling ECM was accompanied by alteration of signals activation in tumor, including focal adhesion kinase, MAP kinases, and the PI3K-AKT cascade (19–21). In this study, our results suggested that 4-OHA could inactivate the PI3K-Akt signaling pathway (**Figure 4C**), which was further confirmed by immunoblotting and immunohistochemistry assays. The results showed that phosphorylated Akt, but not Akt, was significantly reduced by 4-OHA treatment *in vivo* and *in vitro* (**Figures 5C, D, G**; **Supplementary** **Figures 3A, B**). In addition, Akt overexpression in MCF-7 cells can counteract the ability of 4-OHA inhibiting breast cancer growth (**Figures 5H–J**; **Supplementary** **Figure 3C**).

It is well known that cell cycle transitions are regulated by the PI3K-Akt signaling pathway. To confirm the expression levels of cell cycle-related genes and proteins that decreased with the reduction of PI3K-Akt signaling, qRT-PCR and immunoblotting were carried out following 4-OHA treatment. As shown in **Figures 5B–G**, the expression levels of cell-cycle-related genes significantly decreased, while the expression of cyclin kinase inhibitor P27Kip1 was significantly upregulated by 4-OHA. In tumor tissues, the variation of CyclinD1 and P27Kip1 were further verified by immunohistochemistry again (**Figure 5D**).

Above all, results suggested that 4-OHA arrested the cell cycle of breast cancer cells by reduction of PI3K-Akt signaling caused.

### 3.5 4-OHA antitumor growth by upregulating tumor suppressors

As shown in **Figure 4C**, both 4-OHA formulations had a significant impact on axon guidance in mammary tumors. The expression levels of *Lrrc4* and *Sema3b* in axon guidance were significant upregulation *in vivo* and *in vitro* (**Figures 5A, E**). Another two tumor suppressor genes *Btg1* and *Nbl1* were also significantly upregulated following 4-OHA treatment (**Figures 5A, E**).

### 3.6 4-OHA enhanced the infiltration of immune cells into tumor tissues

**Figure 6:**
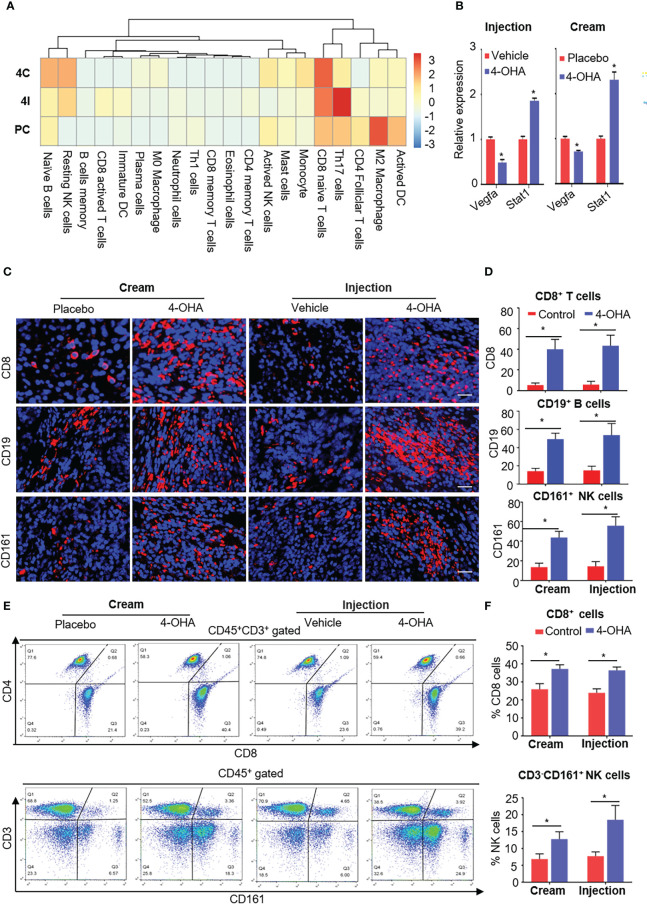
4-OHA treatment remodeled the tumor immune microenvironment. Female SD rats with at least one tumor reaching a size of 1.5 cm in diameter received vehicle peanut oil, placebo cream, 4-OHA cream, or 4-OHA injection for 4 weeks, as indicated in the Materials and Methods. **(A)** Heatmap of relative infiltrations of immune-cell populations in tumor tissues. **(B)** Expression levels of immune-related genes. **(C)** Representative images of tumors stained for CD8, CD19, or CD16/CD56. DAPI (nuclei) was also shown with each antibody. Scale bar, 20 μm. **(D)** Percentage of CD8, CD19, or CD161 positive cells. Counts were quantified from five representative high-powered fields from each sample. **(E, F)** Flow cytometric analysis of the number of CD45+CD3+CD8+ and CD3- CD161+ NK cells in the tumor tissues. E shows an exemplary gating, F gives an overview of the results versus WT. Data are represented as the mean ± SEM. bars, SEM. *p ≤ 0.05 vs. control. PC, Placebo cream; 4C, 4-OHA cream; VI, vehicle; 4I, 4-OHA injection.

It is reported that ECM degradation could improve the infiltration of immune cells into tumor tissues and achieve strong immune response to antitumor immunity (22). As mentioned above, both 4-OHA formulations reduced the components of ECM. To verify whether 4-OHA promoted immune infiltration in DMBA-induced mammary tumors, we performed the CIBERSORT algorithm to calculate the relative proportion of immune cells in tumor tissues treated with/without 4-OHA (**Figure 6A**). The results showed that the proportion of naive B cells, resting NK cells, and naïve CD8^+^ T cells dramatically increased. In contrast, the proportion of M2 macrophages and activated DCs were reduced in both 4-OHA formulations groups as compared with controls. Additionally, several types of immune cells exhibited statistically significant differences between the 4-OHA injection group and the 4-OHA cream group, including activated CD8^+^ T cells, immature DCs, and Th17 cells. They increased in the 4-OHA injection group alone. Furthermore, the expression levels of interferon-signaling genes (e.g., *Stat1, Stat3*) were up-regulated after 4-OHA treatment (**Figure 6B**), while the immunosuppressive cytokine genes *Vegfa* in the 4-OHA cream and injection group displayed 2.04- and 1.39-fold downregulation, respectively (**Figure 6B**).

To verify the results of bioinformatic analysis, we further conducted the immunofluorescence analysis to evaluate the distribution patterns of CD8^+^ T cells, CD19^+^ B cells, and NK cells in tumors treated with 4-OHA or not. Representative tumor sections stained for the CD8, CD19, or CD16/CD56 antibody labeled with dye, and cell nucleus labeled with DAPI are shown in **Figure 6C**. Both formulations of 4-OHA resulted in significantly increasing numbers of tumor-infiltrating CD8^+^ T cells, CD19^+^ B cells, and NK cells as compared with their respective control (**Figure 6D**). The results of flow cytometry analysis also confirmed that CD8^+^ T cells and NK cells play a role in 4-OHA against breast cancer growth (**Figures 6E, F**).

The above results indicated that 4-OHA could significantly promote the infiltration of immune cells into DMBA-induced mammary tumors, but with some differences between the two formulations, such as there were more active CD8^+^ T cells, immature DCs, and Th17 cells in the 4-OHA injection group than in the 4-OHA cream group.

## 4 Discussion

Previously, the potent aromatase inactivator (= suicide inhibitor) 4-OHA was developed by Ciba and Novartis, respectively, as intramuscular depot injection preparation (Lentaron^®^Depot) for treatment of the postmenopausal patients with advanced breast cancer. The medication proved to be very effective and showed high systemic tolerability but was hampered by local tolerability problems, which also did not allow increasing the dosing for higher efficacy. These aspects and the availability of new potent oral aromatase inhibitors led to the strategic decision to stop the marketing of Lentaron^®^Depot by Novartis. However, clinical studies had shown that 4-OHA still worked, when the nonsteroidal AIs failed, which led to a desire to exploit this compound again.

In the late 90s of the last century, researchers at Freiburg postulated that a topical formulation of steroidal aromatase inactivators would be of benefit and could overcome the limitations (local intolerance at higher concentrations) of intramuscular formulation of Lentaron^®^. The novel concept to treat breast cancer with a topical formulation of formestane is based on two rationales: (i) Formestane is by chemical nature a steroid like molecule; this molecule is known to be well resorbed *via* the skin. Estrogen-containing creams are used for a variety of diseases, such as estrogen patches for the treatment of certain menopausal conditions. As Formestane differs only by minor modification from estrogen it should be applied by the transdermal route. (ii) It is well described that breast cancer tissue itself can produce its estrogen by expressing the necessary quantities of the aromatase enzyme. Thus, exposure of the tumor to an aromatase inhibitor *via* topical transdermal application should allow achieving significant inhibitory levels in the tumor being eventually higher than *via* the systemic route. However, so far no literature reported the effects of these new formulations of aromatase inhibitors on breast cancer and their mechanism of action.

In the present study, we developed 4-OHA cream and compared its effect on DMBA-induced mammary tumors with that of 4-OHA subcutaneous injection. Moreover, we used high-throughput sequencing technology to explore the molecular mechanism of action of this cream anti-breast cancer.

Brodie had proved that 4-OHA could reduce ovarian estrogen production and cause regression of carcinogen (DMBA)-induced mammary tumors (9). As shown in **Figure 2A**, we got the same results as Brodie’s when 4-OHA was subcutaneously injected in rats at a dose of 50 mg/kg per day. Of course, this antitumor effect was not due to 4-OHA toxicity, because the toxicity assessment assay showed that 4-OHA cream at the dose was safe (**Figures 3A, B**). The cream absorbed directly *via* the skin might cause a locally high concentration of 4-OHA in the tumor, which efficiently and directly reduced estrogen biosynthesis in tumors and led to an inhibition of tumor growth. Furthermore, analysis of the cell cycles by flow cytometry clearly showed an arrest of breast cancer cells in the G1/G0 phase (**Figures 2H, I**), indicating that the 4-OHA antiproliferative effect on breast cancer cells was due to the cell cycle retardation.

Next, through high-throughput sequencing, we identified the differential expression genes (DEGs) after 4-OHA treatment the DMBA-induced tumors for 28 days. DEGs were primarily associated with extracellular matrix (ECM) ECM-receptor interaction, focal adhesion, PI3K-Akt signaling pathway, axon guidance, and tumor suppressors. Literature reported that estradiol (E2) could increase ECM remodeling, accompanied by upregulating transcripts for *COL1A1*, and several matricellular proteins, including TNC, FN1, and POSTN (19). 4-OHA as a well-known aromatase inhibitor could efficiently reduce estrogen biosynthesis in tumor tissues, which might cause a reduction of ECM remodeling in the tumor microenvironment. As shown in **Figures 5A, E**, 4-OHA decreased the components of ECM in breast cancer cells by downregulating the expression levels of ECM-related genes, such as *Col1a1*, *Tnc*, *Fn1*, and *Itga5*. Moreover, these ECM-related genes play a role in the PI3K-Akt signaling pathway (20, 23, 24). The reduction of phosphorylated AKT, a key protein of the pathway, was determined by Western blot and immunohistochemistry (**Figures 5C, D, G**). These findings indicated that 4-OHA could reduce the PI3K-Akt signaling. And then, the reduction of the PI3K-Akt signaling downregulated the cell cycle-related gene or protein expression (**Figures 5B, C, F, G**), which resulted in the arrest of breast cancer cells in the G1/G0 phase. In addition, the downregulation of ECM-related genes might influence breast cancer metastasis, which would be the topic of future research.

Furthermore, 4-OHA significantly up-regulated the expression levels of tumor suppressors, such as *Lrrc4*, *Sema3b*, *Btg1*, and *Nbl1*. Of these tumor suppressors, *Btg1* had higher expressed levels in the 4-OHA treatment group than the control group (4-OHA cream 798.8 vs. control 297.8; 4-OHA injection 888.1 vs. 328). *BTG1* belongs to the BTG/Tob families, had been proved to inhibit various cancer cells growth, and expresses primarily in the G0/G1 phase transition, with levels decreasing as cells enter S phase (25). Literature also reported that overexpression of *BTG*1 decreased the levels of phosphorylated CDC2, cyclin B1, cyclin D1, and cyclin E1 in the MCF-7 cells (26). As for the two tumor suppressors *Lrrc4* and *Sema3b*, they were rich in the axon guidance signaling pathway. *LRRC4* could arrest the cell cycle in late G1 phase by upregulating the level of cell cycle inhibitory molecules and downregulating the expression of cell cycle regulatory proteins (27–29). Sema3b inhibited axonal extension and exerted an antitumor effect on lung and ovarian cancer cells *in vitro* (30). *Nbl1* also had a work on preventing cells from entering the final stage (G1/S) (31). Therefore, the upregulation of the four tumor suppressors partly accounted for the antitumor activity of 4-OHA cream.

Moreover, increasing evidence indicates that small-molecule inhibitors can remodel the tumor immune microenvironment and improve immune-mediated tumor destruction (32–34). It was reported that the dense ECM blocks the infiltration of immune cells into tumor tissue, limiting the antitumor effect of immunotherapy (35, 36). On the other hand, the degradation of tumor ECM can promote the infiltrations of immune cells into tumors (22). Given these views, 4-OHA also could reduce the components of breast cancer ECM, such as collagens, integrins, and fibronectins (**Figure 4** and **Figure 5**). Hence, 4-OHA increased the number of infiltrated immune cells in DMBA-induced tumors, including naive B cells, resting NK cells, and naïve CD8^+^ T cells, but reduced that of M2 Macrophage and DC Active (**Figure 6A**). Immunofluorescence analysis confirmed the proportion of CD8^+^ T cells, NK cells, and B cells increased in DMBA-induced tumor tissues after treatment with 4-OHA or not (**Figures 6C, D**). The antitumor role of NK cells and cytotoxic T cells has long been well-documented. However, the role of B cells is still controversial in tumor immune microenvironment. Recently, literature reported that tumor-infiltrated B-cells were significantly associated with improved survival in breast cancer patients (37–39). In contrast, M2 macrophages support tumor growth and progression through hypoxia and the secretion of cytokines (e.g. IL-4). Although according to the results of bioinformatic analysis, the 4-OHA treatment decreased the proportion of activated DCs; facilitated the infiltration of naive B cells, resting NK cells, and naïve CD8^+^ T cells into tumor tissue; and did not activate these three immune cells, qRT-PCR results showed that the expression levels of interferon-signaling genes (e.g., *Stat1*) and immunosuppressive cytokine genes (e.g., *Vegfa*) were up- and down-regulated in response to 4-OHA treatments, respectively (**Figure 6B**). This result indicated that 4-OHA could induce a proinflammatory immune response in the tumor microenvironment. Taken together, we concluded that 4-OHA treatment promoted recruitment of lymphocyte in tumor tissue to regulate tumor growth. From this perspective, 4-OHA inducing the lymphocyte infiltration is a promising strategy to convert poorly infiltrated breast cancer from a ‘cold’ tumor to a ‘hot’ tumor.

Additionally, in 4-OHA injection group tumor tissues, there were more active CD8^+^ T cells, immature DCs, and Th17 cells than in 4-OHA cream group tumor tissues, which suggested that there were other mechanisms of action of 4-OHA injection formulation on DMBA-induced of tumors. 4-OHA injection agents might reach multiple organs of rats through the blood circulation system and have complex metabolic pathways and metabolites, which might produce different drug effects in the body of rats, and then promoted the infiltration of more types of immune cells. Whereas, the 4-OHA cream directly infiltrated into the tumor lesion by penetrating the skin without passing through the liver and other organs, which caused a relatively simple metabolic pathway and simpler drug effects.

Moreover, the morphologic changes of 4-OHA cream entering the body might lead to different effects of drugs remodeling the immune environment from 4-OHA injection. This will be the topic of future research.

## 5 Conclusions

This study showed that 4-OHA cream could penetrate the skin and play a role in antitumor action as intramuscular depot preparation (Lentaron^®^Depot). Further, we preliminarily elucidated the molecular mechanism of 4-OHA against breast cancer. The results suggested that Formestane was being reintroduced as a cream for breast cancer therapy. Importantly, the results of the study gives us a way out for new use of old drugs straddling another therapeutic niche, such as atypical immunotherapy like this study.

## Data availability statement

The data presented in the study are deposited in the NCBI databases repository, accession number PRJNA945180. https://www.ncbi.nlm.nih.gov/bioproject/PRJNA945180.

## Ethics statement

The animal study protocol was approved by the Institutional Review Board (or Ethics Committee) of The Affiliated Hospital of Southwest Medical University, Southwest Medical University (protocol code 201903-37 and date of approval 2019-03-05).

## Author contributions

All authors participated in the design, interpretation of the studies, analysis of the data, and review of the manuscript. YL, LG, and WF conceived and designed the whole project and wrote the manuscript. LG and LeZ performed the data analyses. LG, YL, XH, and LeZ interpreted the data and partook in the discussion. CS, YC, LiZ, HQ, and AT revised the final version of the manuscript. All authors contributed to the article and approved the submitted version.
