# Supplementary material for: The transdermal cream of Formestane anti-breast cancer by controlling PI3K-Akt pathway and the tumor immune microenvironment
Source: Front Immunol. 2023 Mar 28;14:1041525. doi: 10.3389/fimmu.2023.1041525 (PMC10087521; doi:10.3389/fimmu.2023.1041525)
Supplement: Supplementary file 1 [file DataSheet_1.docx]

Supplementary Materials


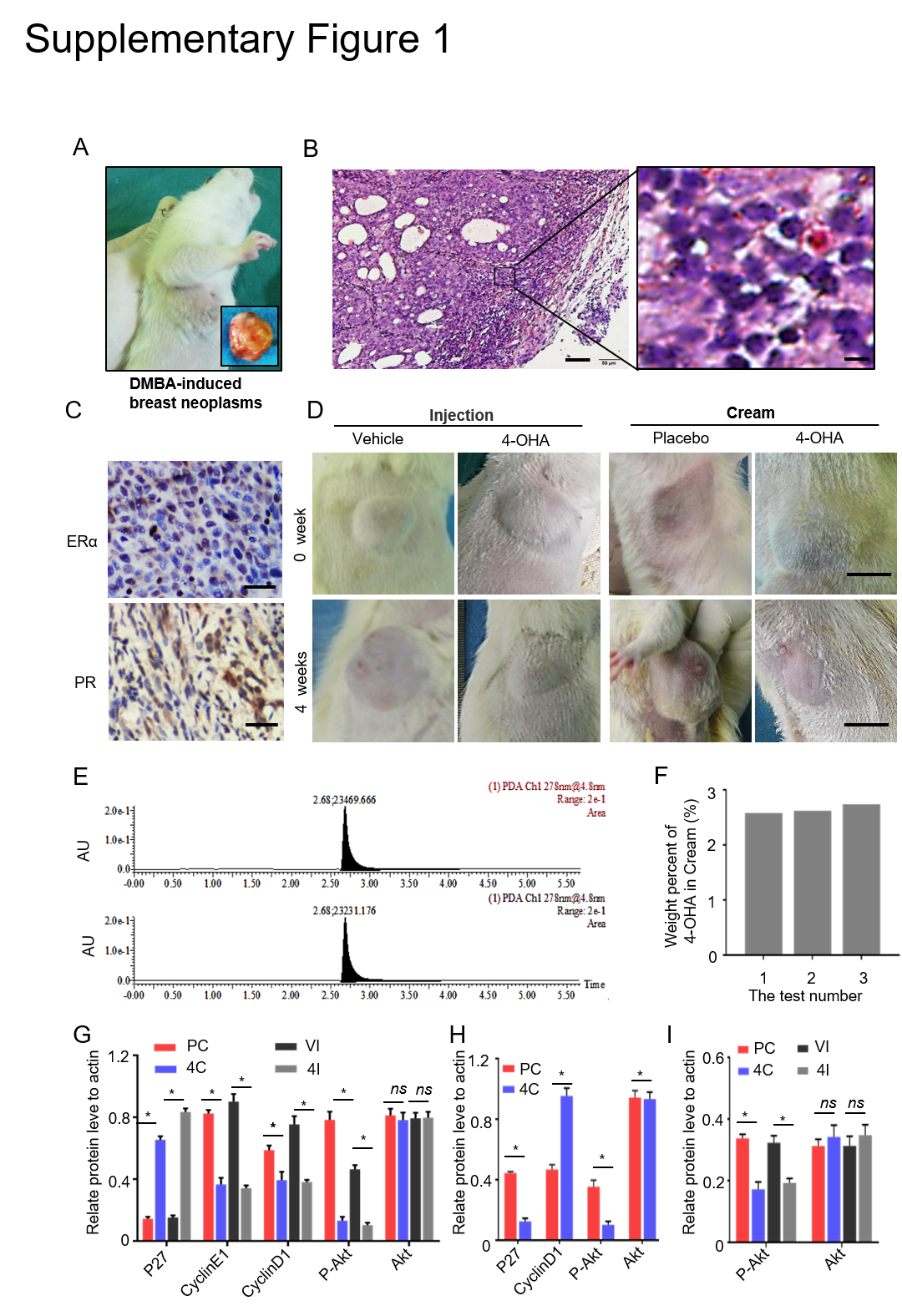


**Supplementary Figure 1 4-OHA cream inhibited DMBA-induced mammary tumor growth. (A)** DMBA-induced mammary tumor. **(B)** H&E staining of mammary tumor induced by DMBA. Scale bar, 50 μm. **(C)** Immunohistochemistry measurement of the expression of estrogen and progesterone receptor in a tumor. Scale bar, 20 μm. **(D)** Representative images of the tumors treated with or without 4-OHA. Scale bar, 10 mm.


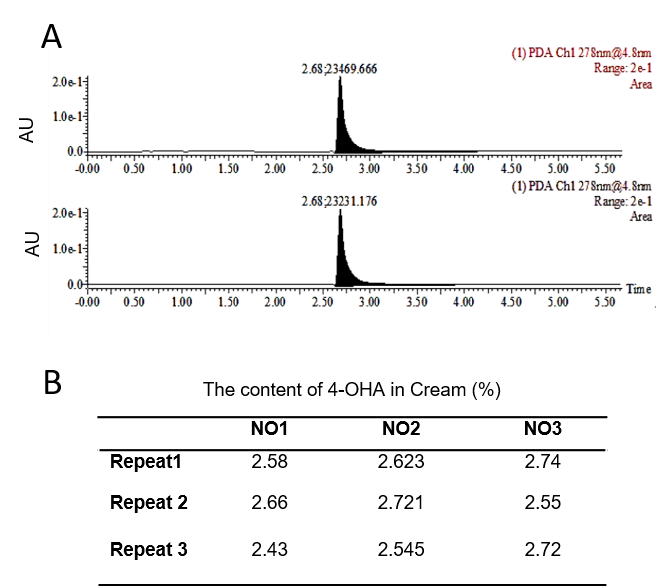


**Supplementary Figure 2** 4-OHA content in cream **(A)** UPLC analysis of 4-OHA content in cream. The peak of a single compound of 4-OHA. **(B)** 4-OHA content of the cream. 4-OHA creams were made at three different time (NO1, NO2, NO3), and the 4OHA content of each cream was repeated three times .


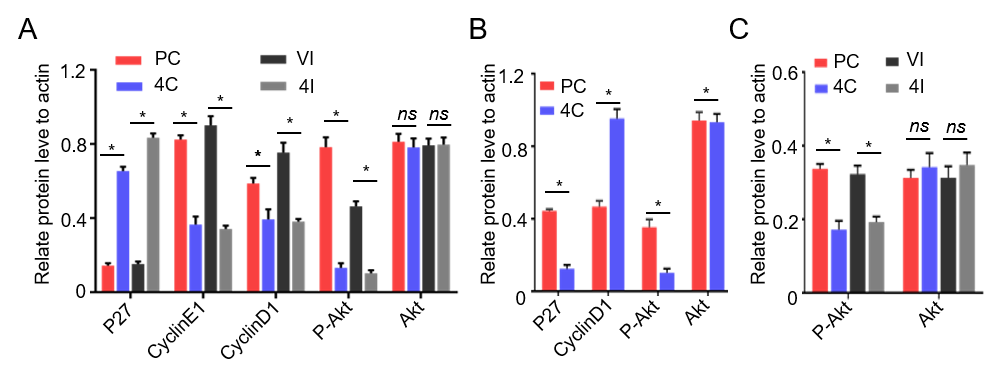


**Supplementary Figure 3** The protein levels was normalized to β-actin. **(A)** The normalized values of protein stips are in figure 5C. **(B)** The normalized values of protein stips are in figure 5D. **(C)** The normalized values of protein stips are in figure 5H. *p <* 0.05, * . *p >* 0.05, *ns.* PC, Placebo cream; 4C, 4-OHA cream; VI, vehicle; 4I, 4-OHA injection.

**Supplementary Table 1** **Sequences of primer in rat**

| **Gene** | **Upstream Primers (5' to 3')** | **Downstream Primers (5' to 3')** |
| --- | --- | --- |
| *lrrc4* | AAAGGGCACAGGCTCCATTTA | AACTCCATGCCCAACTTGTTC |
| *Sema3b* | GGGAAGAGCTGTATTCCGGG | ATCGTCATCAGGGTTCTCGC |
| *Btg1* | GTTACCGTTGTATTCGCATCA | CTGTAGGACACTTCGTAGGGG |
| *Nbl1* | GGCTCGCATCCCACTTTCTA | CCCAAAGCATTGTGCCTGTC |
| *Tnc*-F | CTGAACCACAAGAAATAACCCTC | GAGAACATGATTTCCTTCGGAG |
| *Colla1* | ACCTGTGTGTTCCCCACTCA | AACCTTCGCTTCCATACTCG |
| *Fn1* | CCAACTCATTCACCGTCCAC | AGAATGCTCGGCGTGATG |
| *Itga5* | TCTGGGCCAAGACCTTCT | GGCAGGATTTGGTAGGGCAT |
| *Ccnb1* | GCCTGAGCCTGAACCTGT | CATTGGGCTTGGAGAGGGAG |
| *Ccnd1* | CTACCGCACAACGCACTTTC | AGTCCTTGTTTAGCCAGAGGC |
| *Ccne1* | CTCCGACCTCTCAGTCCGAT | GTGGGGATGAAAGAGCAGGG |
| *Cdk4* | GCCGGAGATGGAGGAATCTG | CGGAAACTGGAAAAGGCAGC |
| *Cdkn1b* | GACTCACTCGCGGCTCC | GGCTCCCGTTAGACACTCTC |
| *Vegfa* | TGTGTGCCCCTAATGCGGT | CGCTCTGAACAAGGCTCACAG |
| *Stat1* | TGGGGCAGAACGAGGTTGTA | ACCTCGATGGGGAAATGCTG |
| *β-actin* | CCCGCGAGTACAACCTTCTT | CGCAGCGATATCGTCATCCA |

**Supplementary Table 2 Sequences of primer in human**

| **Gene** | **Upstream Primers (5' to 3')** | **Downstream Primers (5' to 3')** |
| --- | --- | --- |
| *LRRC4* | CCTGGAGCTGTTCGACAACTG | GAAGGCGTAAGAGGGGATGC |
| *SEMA3B* | ACGTCCAAGTCTCCGAACAGA | GCCGTCTCACGAAAGAAGAAGT |
| *BTG1* | GCACTCAAAACAGCACCAAC | CACCCAAAGCAAAAATCAAA |
| *NBL1* | CATGTGGGAGATTGTGACGCT | CCTCGTGACTAGGCTCCTTG |
| *TNC* | GGTGTTTAACCACGTTTACAA | GGGATGTTGATGCGATGT |
| *COLLA1* | AGTTTTTATCTTTGACCAACCGAA | AAGGGACTTACCCCCGCAT |
| *FN1* | GAACCGGGAACCGAATATAC | AGGGGTCTTTTGAACTGTGG |
| *ITGA5* | GGCTTCAACTTAGACGCGGAG | TGGCTGGTATTAGCCTTGGGT |
| *CCNB1* | AATAAGGCGAAGATCAACATGGC | TTTGTTACCAATGTCCCCAAGAG |
| *CCND1* | GCTGTGTTATTCTTTGCGTGTA | ATGAGAGTCCTACAGGTACAACG |
| *CCNE1* | CAGCGGTTGTAATGTGACCC | AAAGCTCTTCCCCACCCAAT |
| *CDK4* | CTGCAGGCTCATACCATCCT | ACTCTTGAGGGCCACAAAGT |
| *CDKN1B* | GGCTAACTCTGAGGACACGCA | TGGGGAACCGTCTGAAACAT |
| β-actin | GGGAAATCGTGCGTGACATTAAG | TGTGTTGGCGTACAGGTCTTTG |

**Supplementary Table 3 List of all antibodies and sources**

| **Antibody** | **Product code** | **Company** | **Dilution** |  |
| --- | --- | --- | --- | --- |
| β-actin | #58169 | Cell Signaling | 1:5000 |  |
| ERα | #13826 | Cell Signaling | 1:1000 |  |
| PR | 25871-1-AP | proteintech | 1:1000 |  |
| P27^kip^ | 25614-1-AP | proteintech | 1:1000 |  |
| CyclinB1 | #12231 | Cell Signaling | 1:1000 |  |
| CyclinD1 | #2978 | Cell Signaling | 1:1000 |  |
| CyclinE1 | #20808 | Cell Signaling | 1:1000 |  |
| Ki67 | Sc-23900 | Santa Cruz mouse | 1:200 |  |
| AKT | #9272 | Cell Signaling | 1:1000 |  |
| P-Akt1(Ser473) | AF1546 | Beyotime | 1:600 |  |
| Rabbit IgG | AS014 | Abclonal | 1:20000 |  |
| Mouse IgG | AS003 | Abclonal | 1:20000 |  |
| CD8 | #98941 | Cell Signaling | 1:200 |  |
| CD19 | #90176 | Cell Signaling | 1:200 |  |
| CD56/16 | 340300 | BD | 1:200 |  |
| IgG H&L (Alexa Fluor® 594) | ab150088 | Abcam | 1:400 |  |
