# Supplementary material for: The transdermal cream of Formestane anti-breast cancer by controlling PI3K-Akt pathway and the tumor immune microenvironment
Source: Front Immunol. 2023 Mar 28;14:1041525. doi: 10.3389/fimmu.2023.1041525 (PMC10087521; doi:10.3389/fimmu.2023.1041525)
Supplement: Supplementary file 2 [file DataSheet_2.pdf]

Figure 5c

CCND1 P-Akt actin

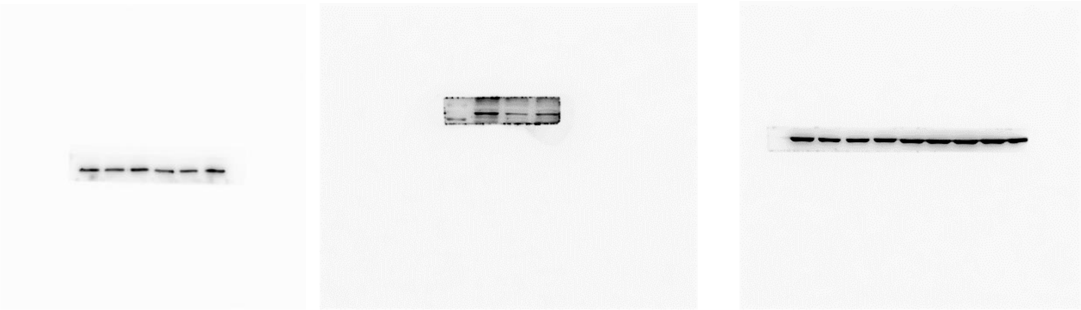

Akt P-Akt Akt

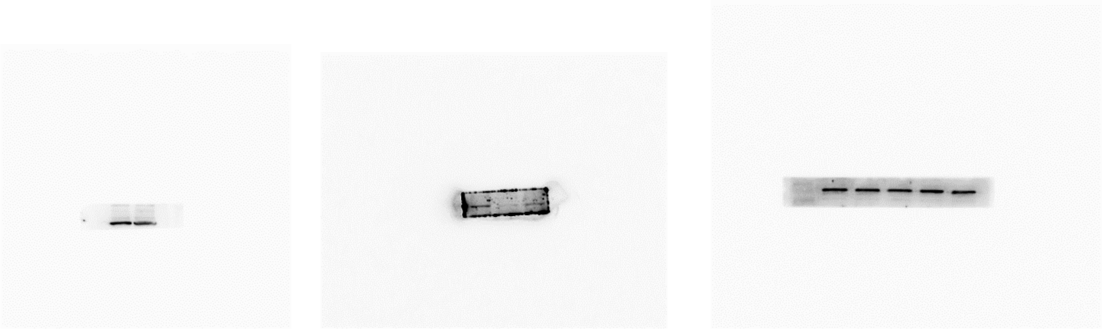

P27 CCNE1

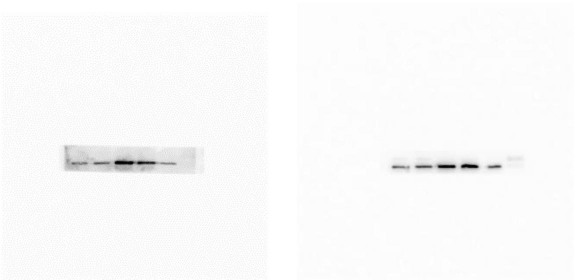

Figure 5D

CCND1

Placebo 4OHA cream Vehicle 4OHA injection

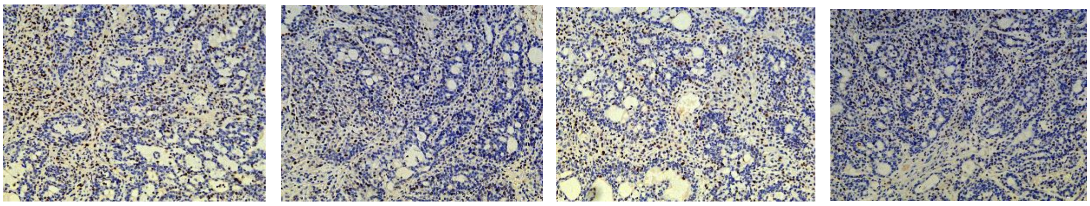

Ki67

Placebo 4OHA cream Vehicle 4OHA injection

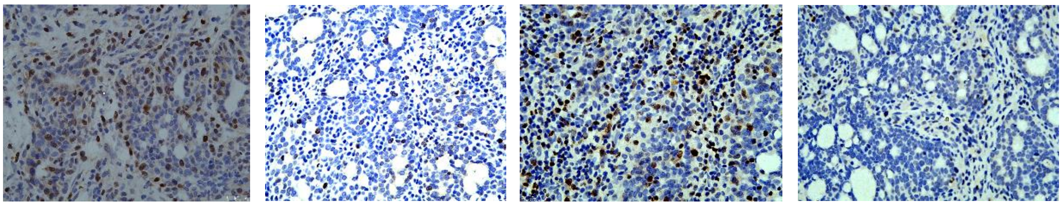

P27

Placebo | 4OHA cream | Vehicle | 4OHA injection

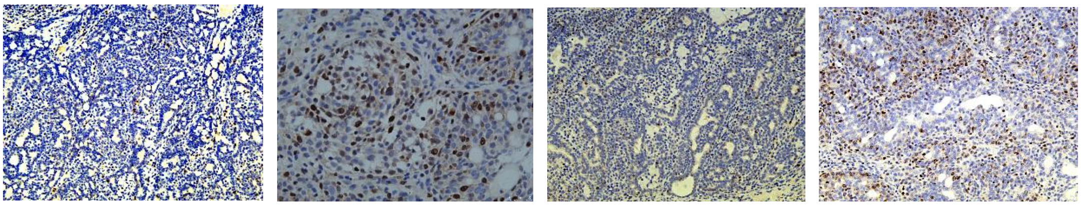

P-Akt

Placebo | 4OHA cream | Vehicle | 4OHA injection

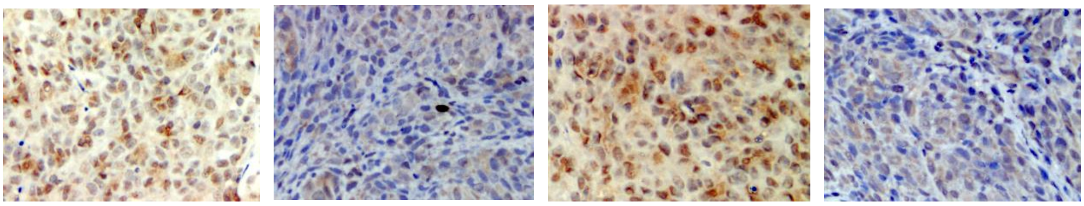

Figure 5G

CCND1 | **actin** | **Akt** | **P-Akt** | **P27**

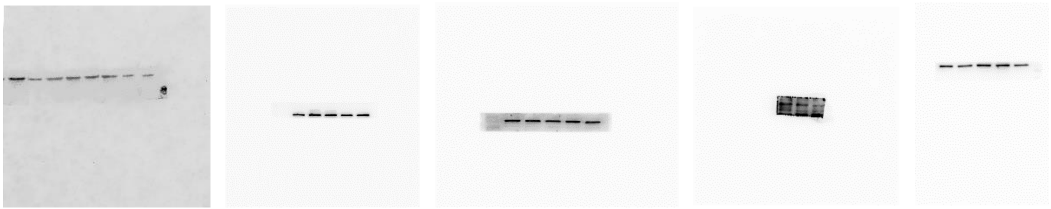

Figure 5H

actin | **Akt** | **p-Akt**

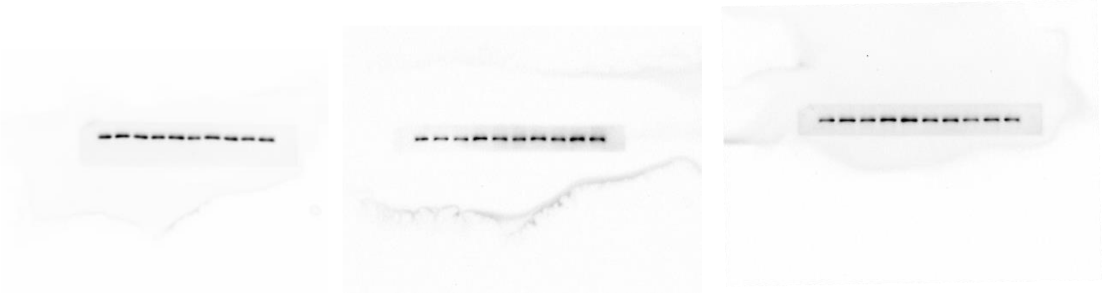

Figure 6C

CD8

Placebo | 4OHA cream | Vehicle | 4OHA injection

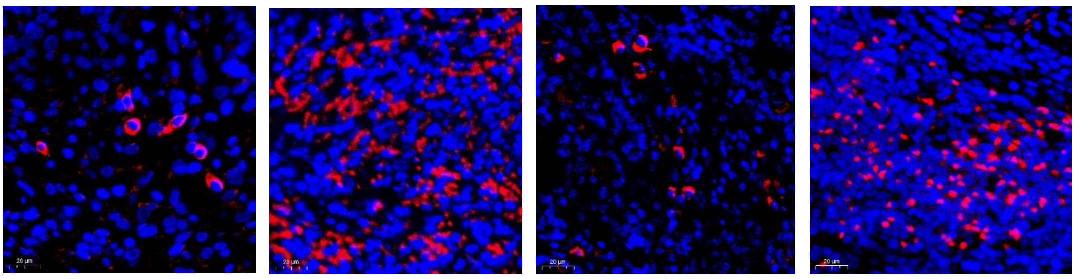

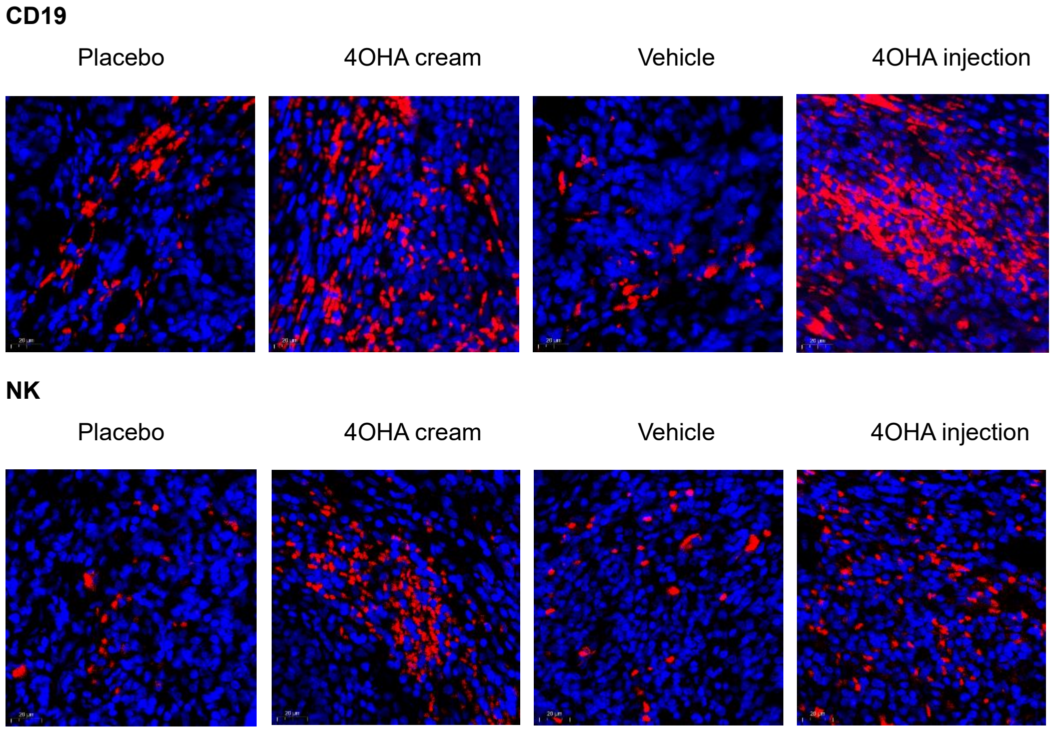
CD19
Placebo
4OHA cream
Vehicle
4OHA injection
NK
Placebo
4OHA cream
Vehicle
4OHA injection
